# Impact of Insomnia on Burnout Among Chinese Nurses Under the Regular COVID-19 Epidemic Prevention and Control: Parallel Mediating Effects of Anxiety and Depression

**DOI:** 10.3389/ijph.2023.1605688

**Published:** 2023-03-16

**Authors:** Xiaofei Mao, Xueru Lin, Peng Liu, Jianguo Zhang, Wenxi Deng, Ziqiang Li, Tianya Hou, Wei Dong

**Affiliations:** ^1^ Faculty of Psychology, Naval Medical University, Shanghai, China; ^2^ Teaching and Research Support Center, Naval Medical University, Shanghai, China; ^3^ Dean’s Office, Naval Medical University, Shanghai, China

**Keywords:** anxiety, burnout, depression, insomnia, regular, COVID-19

## Abstract

**Objective:** To investigate the mediating effects of anxiety and depression in the relationship between insomnia and burnout among Chinese nurses under the regular COVID-19 epidemic prevention and control.

**Methods:** Convenience sampling was applied to recruit 784 nurses in Jiangsu Province, China. The respondents completed the survey *via* mobile devices. Demographic questionnaire, Insomnia Severity Index, Generalized Anxiety Disorder-7, Patient Health Questionnaire-9 and Maslach Burnout Inventory were used to assess demographic information, insomnia, anxiety, depression, and burnout, respectively. Hayes PROCESS macro was employed to examine the mediation model.

**Results:** Insomnia, anxiety, depression and burnout were positively and significantly associated with each other. Anxiety and depression played partial mediation effects between insomnia and burnout with the mediation effect of anxiety and depression accounting for 28.87% and 31.69% of the total effect, respectively.

**Conclusion:** Insomnia may lead to burnout through the parallel mediating effects of anxiety and depression in Chinese nurses. Interventions on sleep, anxiety and depression from the hospital management were essential to ameliorate nurses’ burnout status under the regular COVID-19 epidemic prevention and control.

## Introduction

Burnout, a syndrome of emotional exhaustion and cynicism, often happens to individuals who are engaged in some kinds of “people-work” (1), and it is often measured using the Maslach Burnout Inventory with 3 dimensions (emotional exhaustion, depersonalization and personal accomplishment) (2). Nurses are more likely to have job burnout, since they work in different conditions of clinical settings and are close related with patient care, which puts high physical and psychological pressure on nurses (3). Literatures showed insomnia and burnout were closely related with each other in nurses (4–8).

Insomnia and burnout in nurses during COVID-19 pandemic drew plenty of attention from researchers. Aydin Sayilan et al. reported that nurses working in the pandemic of COVID-19 were determined to be at risk of insomnia and burnout, they also found a statistically significant positive association of sleep quality with emotional exhaustion and depersonalization scores (9). Another research by Dos Santos et al. indicated that the incidence of sleep disorders and burnout were prevalent among nursing professionals during the COVID-19 pandemic, nurses with sleep disorders presented a high or moderate level of emotional exhaustion and a high level of burnout related to personal accomplishment (10).

According to studies based on individuals in the workforce, there may exist a causal relationship between insomnia and burnout. For example, Jansson-Fröjmark and Lindblom employed a prospective design in 2010 and found insomnia was linked to the maintenance of the central part of burnout, while burnout was not related to future insomnia (11). Söderström et al. also designed a prospective study in 2012 and discovered that insufficient sleep was risk factor for subsequent burnout (12). Thus, it could be inferred that insomnia may lead to burnout in nurses.

Many studies were conducted to explore the association of insomnia with anxiety and depression. Individuals with clinical insomnia had a higher risk of developing anxiety and depression than those without insomnia (13, 14). Specifically, participants with insomnia were 17.35 and 9.82 times as likely to have anxiety and depression than those without insomnia, respectively (15). These studies suggested insomnia may have a negative effect on depression and anxiety.

Anxiety and depression were vital factors affecting burnout of nurses in the period of COVID-19. Noh et al. discovered depression and anxiety were positively correlated with burnout. Moreover, Noh et al. revealed depression was a major factor influencing burnout of frontline nurses by hierarchical regression analysis during the COVID-19 pandemic in South Korea (16). Liu and Zhang found positive correlations between burnout, anxiety and depression among Chinese nurses in the period of COVID-19. What’s more, they found anxiety could positively predict burnout by multiple stepwise regression analysis (17).

Therefore, we could infer that insomnia may lead to burnout among nurses in the period of COVID-19. In addition, anxiety and depression were important influencing factors in the relationship between insomnia and burnout.

With the effort of Chinese government, the epidemic situation of COVID-19 has been effectively controlled. China entered the stage of normalized epidemic prevention and control since May 2020 (18). Though the severe situation faced by nurses has changed, they still worked under great pressure, such as wearing protective clothing, having virus nucleic acid test and conducting chest X-ray inspection for hospitalized patients. For example, Chen et al. investigated the mental health status of nurses under the normalized COVID-19 pandemic prevention and control in China, and found 22.0%, 29.8% and 16.1% of them reported moderate and extreme levels of depression, anxiety and stress, respectively (19). It is important to understand the relationships among insomnia, anxiety, depression and burnout of Chinese nurses under the regular COVID-19 epidemic prevention and control. Exploring the roles of anxiety and depression in the relationship between insomnia and burnout is practically helpful. It may inform health professionals about with potential measures to reduce or even prevent burnout among nurses during the normalisation of COVID-19 epidemic prevention and control, such as improving nurses’ sleep quality and intervening their anxiety and depression. However, the studies concerning the associations among these variables are still limited.

**FIGURE 1:**
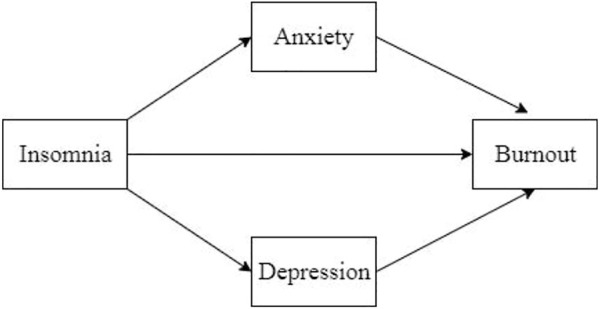
Conceptual framework of hypothesized model. China, 2022.

Therefore, we supposed that anxiety and depression may serve as mediators in the association between insomnia and burnout among nurses during the normalisation of COVID-19 epidemic prevention and control. In the present study, a parallel mediating model was built to classify the mediating effects of anxiety and depression in the relationship between insomnia and burnout among Chinese nurses under the regular COVID-19 epidemic prevention and control. A hypothesized model is presented in Figure 1, which identifies the relationships among insomnia, anxiety, depression and burnout.

## Methods

### Participants and Procedures

A cross-sectional study was conducted in January 2022 among tertiary hospitals. G^*^Power software version 3.1.9.7 was used to estimate the required sample size of this study. The present study used binary logistic regression analysis to analyze the association between mental health and associated factors. Therefore F-test (Linear multiple regression: Fixed model, *R* ^*2*^ increase) was employed. Effect size (*f* ^*2*^) was set at 0.15 and alpha value was set at 0.05. Approximately 189 participants would provide 95.07% power to detect a statistical significance.

There were 3 inclusion criteria in this study. They were listed as follows, I. ability of reading and writing, II. 18 years old or above, and III. working in hospital during the normalisation of COVID-19 epidemic prevention and control. The exclusion criterion was that female nurses had a history of mental illnesses. Convenience sampling was applied to recruit 784 nurses in Jiangsu Province, China. The nurses filled out all the scales in a Chinese version of questionnaire website called Wenjuanxing (https://www.wjx.cn/). The questionnaires can only be submitted after all the questions have been answered. The respondents completed the survey with mobile devices. Ethical approval was obtained from the Naval Medical University before the initiation of the research project. Prior to the online survey, informed written consent was given by all participants. Participants were assured their responses were anonymous and confidential. Participants were free to withdraw at any time without penalty.

### Measures

#### Demographics

In the present study, demographic information including age, gender, years of working, marital status (single or married) were recorded.

#### Insomnia Severity Index, ISI

ISI is a brief self-assessment tool, which has been previously proven as a reliable and valid instrument to quantify perceived insomnia severity (20). It includes 7 items, and each item is rated using a 5-point Likert scale, ranging from 0 (never) to 4 (almost always). A higher score indicates a higher severity of insomnia. The total score ranges from 0 to 28. In the present study, the Cronbach’s alpha was 0.927.

#### Generalized Anxiety Disorder-7, GAD-7

The GAD-7 is a valid and efficient tool for screening anxiety and assessing its severity in clinical practice and research (21). The 7-item questionnaire was used to ask participants how often they were bothered by each symptom during the last 2 weeks. Response options were “not at all” “several days” “more than half the days” and “nearly every day” scored as 0, 1, 2, and 3, respectively. In the present study, the Cronbach’s alpha was 0.960.

#### Patient Health Questionnaire-9, PHQ-9

The PHQ-9 includes 9 items pertaining to the DSM-IV criteria for depressive disorder (22). Each item is rated on a 4-point Likert scale from 0 to 3 (0-never; 1-several days; 2-more than half the time; and 3-nearly every day) within the last 2 weeks before the completion of the survey. In the present study, the Cronbach’s alpha was 0.935.

#### Maslach Burnout Inventory, MBI

The 22-item MBI was used to ask the respondents’ specific feelings related to their work on a 7-point Likert scale with 0 representing “never” and 6 representing “everyday” (1). The MBI consisted of 3 dimensions: emotional exhaustion (EE, 9 items), depersonalization (DP, 5 items), and personal accomplishment (PA, 8 items). In the present study, the Cronbach alpha were 0.902, 0.918, 0.916 and 0.912 for the total and sub-scales, respectively.

### Statistical Analysis

Data were analysed with IBM SPSS (Version 21.0) and PROCESS (Version 3.4.1) macro for SPSS. First of all, Harman single factor test was conducted to examine common method bias. Second, Pearson’s correlation analyses were conducted to investigate the bivariate correlations between the variables. Third, the bootstrapped confidence interval estimates of the indirect effects were analysed to confirm the significance of mediations (23). The bootstrap estimates were calculated based on 5,000 resamples and 95% confidence intervals. The mediating effect was established if 0 was not included in the confidence interval. The significance level was set at *α* = 0.05, and all tests were 2-tailed.

## Results

### Demographic Characteristics

A total of 784 nurses were recruited in the present study. In terms of demographic characteristics, the average age was 30.38 ± 6.60 years old, 44 (5.6%) of the respondents were male, 496 (63.3%) nurses were married. Besides, the average year of working experience were 8.81 ± 6.87 years.

### Common Method Bias Test

In the present study, self-report inventory was adopted to collect data, which may result in a common method bias (24). Therefore, we conduct the Harman single factor test to examine the common method bias (25, 26). The KMO value was 0.95 (*p* < 0.001), indicating that the data in this study were suitable for exploratory factor analysis. There were 6 values with eigenvalue more than 1 and the first factor presented a variance of 36.81% (lower than the criterion of 40%). Therefore, the results showed that there was no serious common method bias problem in this research.

### Bivariate Analysis

The descriptive statistics and correlations among insomnia, anxiety, depression and burnout were listed in Table 1. The results showed that all variables of interest were positively and significantly associated with each other (all *p* < 0.01).

**TABLE 1:** Descriptive statistics and correlations among variables (N = 826). China, 2022.

Variables	Mean ± SD	Insomnia	Anxiety	Depression	Burnout
Insomnia	6.31 ± 5.27	1			
Anxiety	3.62 ± 4.06	0.436**	1		
Depression	6.14 ± 5.15	0.524**	0.821**	1	
Burnout	52.41 ± 18.67	0.383**	0.526**	0.541**	1

**p < 0.01.

### Mediating Roles of Anxiety and Depression

**FIGURE 2:**
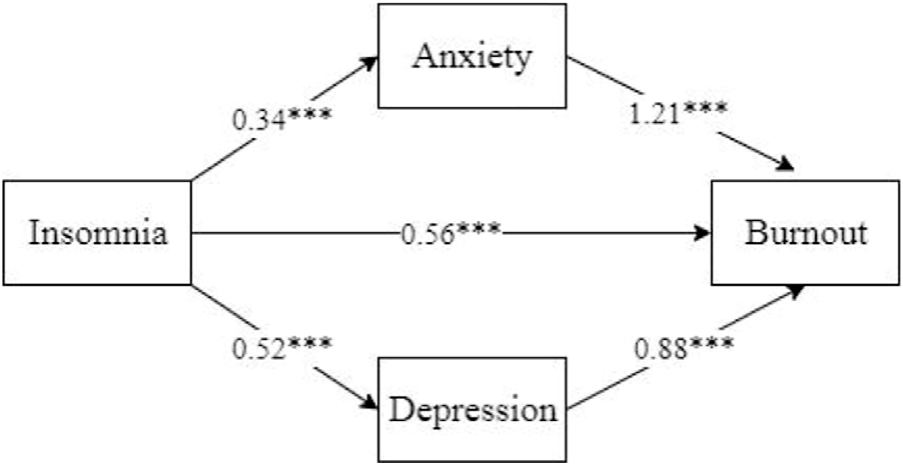
Parallel mediating effects of anxiety and depression on insomnia and burnout (***p < 0.001). China, 2022.

Studies focusing on nurses’ burnout during COVID-19 revealed age (9, 27), gender (9, 16, 25), marital status (9) and years of working experience (17) were closely related with burnout. Therefore, analysis was performed by PROCESS 3.4.1 (Model 4) (28) after controlling the confounders mentioned above with insomnia as independent variable, depression and anxiety as the mediating variables, and burnout as the dependent variable. The Parallel mediating model was presented in Figure 2.

The results of the mediating effects of anxiety and depression were presented in Table 2. The direct effect of insomnia on burnout value was 0.56 [95% CI = (0.33–0.80)], suggesting the direct effect was significant since the 95% confidence interval did not contain 0. The total indirect effect value of anxiety and depression was 0.86 [95% CI = (0.70–1.04)], indicating that the total indirect effect was significant as 95% corrected confidence interval did not include 0. The total mediating effect accounted for 60.56% of the total effect.

**TABLE 2:** Mediating effects of anxiety and depression in the relationship between insomnia and burnout. China, 2022.

	Path	Effect value	Effect size	95% CI	
				LLCI	ULCI
Mediating effects	Insomnia→ Anxiety→ Burnout	0.41	28.87%	0.26	0.57
	Insomnia→ Depression→ Burnout	0.45	31.69%	0.25	0.67
Direct effect	Insomnia→ Burnout	0.56	—	0.33	0.80
Total mediating effect		0.86	60.56%	0.70	1.04
Total effect		1.42	—	1.20	1.65

The indirect effect value of insomnia on burnout *via* anxiety was 0.41 [95% CI = (0.26–0.57)], indicating that the indirect effect of anxiety was significant as 95% corrected confidence interval did not include 0. The mediation effect accounted for 28.87% of the total effect. Besides, the indirect effect value of insomnia on burnout *via* depression was 0.45 [95% CI = (0.25–0.67)], indicating that the indirect effect of depression was significant since 0 was not included in the 95% confidence interval. The ratio of the indirect effect of the total effect was 31.69%.

Consequently, anxiety and depression played partial mediation effects in the association between insomnia and burnout. Specifically, insomnia not only directly affected burnout of nurses, but also resulted in burnout by increasing the level of anxiety and depression among the nurses during the normalisation of COVID-19 epidemic prevention and control.

## Discussion

In the present study, we investigated the parallel mediating roles of anxiety and depression in the relationship between insomnia and burnout among Chinese nurses under the regular COVID-19 epidemic prevention and control. To the best of our knowledge, this is the first study concerning the impact of insomnia upon burnout among Chinese nurses *via* the parallel mediating effects of anxiety and depression.

As hypothesized, anxiety and depression partially mediated the association between insomnia and burnout. First of all, the results showed that insomnia could positively predict burnout, which was consistent with previous literature (11, 12). Scholars from Sweden drew a conclusion from a prospective study focusing employees from IT-company that insufficient sleep was a risk factor for subsequent burnout, and interventions to enhance sleep could help prevent burnout (12). Furthermore, Jansson-Fröjmark and Lindblom conducted a prospective study over a year among individuals in the Swedish workforce and found insomnia was linked to the maintenance of the central part of burnout, while burnout was not related to future insomnia (11). There were also a lot of cross-sectional studies focusing on the relationship between insomnia and burnout in nurses, which indicated insomnia was a significant predictive factor of burnout (29–31). For example, Song et al. found poor sleep quality exhibited strong positive associations with job burnout among Chinese nurses through SEM analysis (29). In addition, research based on nurses who treated patients diagnosed with COVID-19 revealed sleep disturbances posed a significant mediating effect on the relationship between psychological distress and burnout, which indicated sleep problems may affect burnout of nurses (32).

Second, anxiety and depression were found to be the mediators between insomnia and burnout, which highlighted the importance of negative emotions to burnout among Chinese nurses with sleep problems under the regular COVID-19 epidemic prevention and control. The longitudinal analyses from the study of Jansson-Fröjmark and Lindblom (11) demonstrated that anxiety and/or depression were related to the incidence of burnout. Emotional exhaustion (lack of energy and a sense that emotional resources have been exhausted) was the core element of burnout (33). These may be the possible explanations of predictive relationship between negative emotions and burnout in nurses. Besides, insomnia was a strong risk factor of anxiety and depression (34). It can be seen from the above that insomnia could predict burnout of Chinese nurses *via* the mediating roles of anxiety and depression under the regular COVID-19 epidemic prevention and control.

As a whole, this model provided a pathway of the relationship among insomnia, anxiety, depression and burnout of Chinese nurses under the regular COVID-19 epidemic prevention and control. Namely, sleep problems may lead to burnout through the parallel mediating effects of anxiety and depression. Our results not only revealed the possible mechanisms underlying the relationship between insomnia and burnout, but it also provided practical interventions to promoting burnout problem in Chinese nurses under the regular COVID-19 epidemic prevention and control.

Moreover, though China’s prevention and control policies and the status of the pandemic development are completely different from January 2022, the present study still had implications for the current and the future management of nurses’ mental health. First, the present study provided a theoretically grounded foundation for an in-depth understanding of the association between insomnia and burnout and its underlying mechanisms among Chinese nurses. Second, the results could also provide hospital management with potential measures to prevent burnout during public health emergencies or in normal time. Interventions that improve sleep quality should be designed to prevent and mitigate burnout of Chinese nurses. Besides, program focusing on negative emotions should be developed to Chinese nursing staff.

Several limitations in current study need to be mentioned. First of all, the cross-sectional design employed in the present study cannot confirm the causal relationship between insomnia, anxiety, depression and burnout. It is just as likely that burnout could lead to increased anxiety and depression and increased insomnia as the direction proposed in the present study. It remains elusive whether insomnia influences anxiety, depression and burnout or vise versa, or whether they influence each other mutually. Future researchers could employ a longitudinal study design to verify the mediation model. Second, subjects of the present study were all from Jiangsu Province, which may restrict the generalization of our results to other areas of China. Third, our respondents completed the self-reported survey with mobile devices, which might lead to self-reported biases and social desirability response bias. Fourth, years of education, location of nurses (rural and urban areas) and experience in infectious diseases were also potential confounders of the relationship among insomnia, anxiety, depression and burnout, researchers should consider them in the future studies.

### Conclusion

In summary, this study presented parallel mediating effects of anxiety and depression in the association between insomnia and burnout among Chinese nurses under the regular COVID-19 epidemic prevention and control. Namely, insomnia affects burnout of nurses *via* anxiety and depression. Hospital management department should pay special attention to nurses with sleep problems to prevent and reduce burnout under the regular COVID-19 epidemic prevention and control. Moreover, measures targeting the prevention of negative emotions need to be taken to prevent burnout of nurses.
