# Supplementary material for: Impact of Insomnia on Burnout Among Chinese Nurses Under the Regular COVID-19 Epidemic Prevention and Control: Parallel Mediating Effects of Anxiety and Depression
Source: Int J Public Health. 2023 Mar 16;68:1605688. doi: 10.3389/ijph.2023.1605688 (PMC10060543; doi:10.3389/ijph.2023.1605688)
Supplement: Supplementary file 1 [file DataSheet1.docx]

**Demographics Questions**

**Below is a list of demographics questions. Please read each item and fill in.**

1.Age: ( )

2.Gender: ( )

3.Years of working: ( )

6.Marital status: ( )

**Insomnia Severity Index (ISI)**

**Please rate the current Severity of your insomnia problems. (Use “√” to indicate your answer)**

**1.Difficulty falling asleep?**

0-None 1-Mild 2-Moderate 3-Severe 4-Very Severe

**2.Difficulty staying asleep?**

0-None 1-Mild 2-Moderate 3-Severe 4-Very Severe

**3.Problem waking up too early?**

0-None 1-Mild 2-Moderate 3-Severe 4-Very Severe

**4. How SATISFIED/DISSATISFIED are you with your current sleep pattern?**

0-Very satisfied 1-Satisfied 2-Moderate satisfied 3-Dissatisfied 4-Very dissatisfied

**5. To what extent do you consider your sleep problem to INTERFERE with your daily functioning (e.g. daytime fatigue, mood, ability to function at work/daily chores, concentration, memory, mood, etc.) currently?**

0-Not at all interfering 1-A little 2-Somewhat 3-Much 4-Very much interfering

**6.** **How NOTICEABLE to others do you think your sleep problem is in terms of impairing the quality of your life?**

0-Not at all Noticeable 1-Barely 2-Somewhat 3-Much 4-Very much noticeable

**7.How WORRIED/DISTRESSED are you about your current sleep problem?**

0-Not at all worried 1-A little 2-Somewhat 3-Much 4-Very much worried

**Generalized Anxiety Disorder-7**

**Over the last 2 weeks, how often have you been bothered by the following problems? (Use “√” to indicate your answer)**

**1. Feeling nervous, anxious or on edge.**

0-not at all 1-several days 2-more than half the days 3-nearly everyday

**2. Not being able to stop or control worrying.**

0-not at all 1-several days 2-more than half the days 3-nearly everyday

**3. Worrying too much about different things.**

0-not at all 1-several days 2-more than half the days 3-nearly everyday

**4. Trouble relaxing.**

0-not at all 1-several days 2-more than half the days 3-nearly everyday

**5. Being so restless that it is hard to sit still.**

0-not at all 1-several days 2-more than half the days 3-nearly everyday

**6. Becoming easily annoyed or irritable.**

0-not at all 1-several days 2-more than half the days 3-nearly everyday

**7. Feeling afraid as if something awful might happen.**

0-not at all 1-several days 2-more than half the days 3-nearly everyday

**The Patient Health Questionnaire-9 (PHQ-9)**

**Over the last 2 weeks, how often have you been bothered by the following problems? (Use “√” to indicate your answer)**

**1. Little interest or pleasure in doing things.**

0-not at all 1-several days 2-more than half the days 3-nearly everyday

**2. Feeling down, depressed, or hopeless.**

0-not at all 1-several days 2-more than half the days 3-nearly everyday

**3. Trouble falling or staying asleep, or sleeping too much.**

0-not at all 1-several days 2-more than half the days 3-nearly everyday

**4. Feeling tired or having little energy.**

0-not at all 1-several days 2-more than half the days 3-nearly everyday

**5. Poor appetite or overeating.**

0-not at all 1-several days 2-more than half the days 3-nearly everyday

**6. feeling bad about yourself — or that you are a failure or have let yourself or your family down.**

0-not at all 1-several days 2-more than half the days 3-nearly everyday

**7. Trouble concentrating on things, such as reading the newspaper or watching television.**

0-not at all 1-several days 2-more than half the days 3-nearly everyday

**8.Moving or speaking so slowly that other people could have noticed? Or the opposite — being so fidgety or restless that you have been moving around a lot more than usual.**

0-not at all 1-several days 2-more than half the days 3-nearly everyday

**9.Toughts that you would be better off dead or of hurting yourself in some way.**

0-not at all 1-several days 2-more than half the days 3-nearly everyday

**Maslach Burnout Inventory, MBI**

**Over the last month, how often have you been bothered by the following problems? Please read each item and fill your answer in the bracket. (0 representing “never” and 6 representing “everyday”)**

0-never 1-several times a year 2-once a month 3-several times a month

4-once a week 5- several times a week 6-everyday

**Ⅰ. Emotional Exhaustion**

1. I feel emotionally drained from my work. ( )
2. I feel used up at the end of the workday. ( )
3. I feel fatigued when I get up in the morning and have to face another day on the job. ( )
4. Working with people all day is really a strain for me. ( )
5. I feel burned out from my work. ( )
6. I feel frustrated by my job. ( )
7. I feel I'm working too hard on my job. ( )
8. Working with people directly puts too much stress on me. ( )
9. I feel like I'm at the end of my rope. ( )

**II. Personal Accomplishment**

1. I can easily understand how my recipients feel about things. ( )
2. I deal very effectively with the problems of my recipients. ( )
3. I feel I'm positively influencing other people's lives through my work. ( )
4. I feel very energetic. ( )
5. I can easily create a relaxed atmosphere with my recipients. ( )
6. I feel exhilarated after working closely with my recipients. ( )
7. I have accomplished many worthwhile things in this job. ( )
8. In my work, I deal with emotional problems very calmly. ( )

**Ⅲ. Depersonalization**

1. I feel I treat some recipients as if they were impersonal 'objects'. ( )
2. I've become more callous toward people since I took this job. ( )
3. I worry that this job is hardening me emotionally. ( )
4. I don't really care what happens to some recipients. ( )
5. I feel recipients blame me for some of their problems. ( )
